# Regulation of T Cell Differentiation and Alloimmunity by the Cyclin-Dependent Kinase Inhibitor p18ink4c

**DOI:** 10.1371/journal.pone.0091587

**Published:** 2014-03-10

**Authors:** Emily A. Rowell, Liqing Wang, Neelanjana Chunder, Wayne W. Hancock, Andrew D. Wells

**Affiliations:** 1 Department of Pathology and Laboratory Medicine, Perelman School of Medicine at the University of Pennsylvania, Philadelphia, Pennsylvania, United States of America; 2 Department of Pathology and Laboratory Medicine, The Children’s Hospital of Philadelphia Research Institute, Philadelphia, Pennsylvania, United States of America; Northwestern University Feinberg School of Medicine, United States of America

## Abstract

Cellular proliferation in response to mitogenic stimuli is negatively regulated by the Cip/Kip and the Ink4 families of cyclin-dependent kinase (CDK) inhibitors. Several of these proteins are elevated in anergic T cells, suggesting a potential role in the induction or maintenance of tolerance. Our previous studies showed that p27^kip1^ is required for the induction of T cell anergy and transplantation tolerance by costimulatory blockade, but a role for Ink4 proteins in these processes has not been established. Here we show that CD4+ T cells from mice genetically deficient for p18^ink4c^ divide more rapidly than wild-type cells in response to antigenic, costimulatory and growth factor signals. However, this gain of proliferative function was accompanied by a moderate increase in the rate of cell death, and was accompanied by an overall defect in the generation of alloreactive IFNγ-producing effector cells. Consistent with this, p18^ink4c^-deficient T cells were unable to induce graft-vs-host disease *in vivo*, and p18^ink4c^ deficiency cooperated with costimulatory blockade to significantly increase the survival of fully mismatched allografts in a cardiac transplantation model. While both p18^ink4c^ and p27^kip1^ act to restrict T cell proliferation, p18^ink4c^ exerts an opposite effect from p27^kip1^ on alloimmunity and organ transplant rejection, most likely by sustaining T cell survival and the development of effector function. Our studies point to additional important links between the cell cycle machinery and the processes of T cell differentiation, survival and tolerance.

## Introduction

Cyclin-dependent kinase inhibitors negatively regulate the cell cycle, helping to set the threshold for cell cycle entry and promoting exit from cell cycle in response to growth factor withdrawal, inhibitory cytokines, contact inhibition, DNA damage, and senescence. Over-expression of these factors can impose cell cycle arrest in a retinoblastoma protein (pRb)-dependent manner, while elimination of CDK inhibitors leads to enhanced proliferative responses in many types of tissues and cell types. CDK inhibitors are subdivided into two groups based on functional and structural differences. The Cip/Kip family consists of p21^cip1^, p27^kip1^ and p57^kip2^, which share two N-terminal subdomains used to bind to and inhibit multiple cyclin/CDK complexes, including cyclin D-CDK4, cyclin D-CDK6, cyclin E-CDK2 and cyclin A-CDK2 [1]. The Ink4 family consists of four members, p15^ink4b^, p16^ink4a^, p18^ink4c^ and p19^ink4d^, that contain four to five ankyrin repeats and specifically inhibit CDK4 and CDK6 [2]–[6].

Mice deficient for p18^ink4c^ or p27^kip1^ exhibit multi-organ hyperplasia, gigantism, and have abnormally large thymi and secondary lymph nodes, while mice deficient in p15^ink4b^ or p16^ink4a^ exhibit hyperplasia that is largely limited to lymphoid compartments (reviewed in [7]). The enlarged lymphoid compartments of p18^ink4c^- and p27^kip1^-deficient mice are due to increased generation of naïve T cells, as spontaneous accumulation of activated T lymphocytes in the periphery is not observed. p27^kip1^ is an established, intracellular sensor of costimulatory and growth factor signals in both CD4+ and CD8+ T cells [8]–[14], where it limits clonal expansion. p27 is also necessary for the induction of anergy *in vitro* [13], and is required for the induction of immune tolerance to alloantigens *in vivo* [15], [16]. p18^ink4c^ is expressed in naive T lymphocytes and its levels are maintained following activation. Previous studies showed that p18^ink4c^-deficient T lymphocytes exhibit increased CDK6 activity and enhanced proliferative responses to mitogenic stimuli [17], suggesting that p18^ink4c^ may also an important negative regulator of T lymphocyte responses. However, whether p18^ink4c^ contributes to T cell differentiation and function is not known.

In this study, we have utilized p18^ink4c^−/− mice to determine whether p18^ink4c^ affects the threshold for costimulatory and growth factor receptor signaling in T cells, and to determine the role of p18^ink4c^ in the induction of anergy and tolerance. We found that p18^ink4c^ negatively regulates progression from G_0_ into S phase during the first mitosis after activation, but does not regulate subsequent cell divisions during clonal expansion. However, unlike T cells deficient for p27^kip1^, p18^ink4c^−/− T cells exhibit an increased rate of cell death and exhibit a defect in the production of differentiated cytokines such as IFNγ. Consequently, T cells lacking p18^ink4c^ failed to induce graft-vs.-host disease (GVHD) in fully MHC-mismatched recipients, and costimulatory blockade was much more effective at preventing cardiac allograft rejection in recipient mice lacking p18^ink4c^.

## Materials and Methods

### Cell Culture and Reagents

Spleen and lymph node cell suspensions were prepared and labeled with CFSE (Molecular Probes) as described previously [13]. Cells were cultured in medium (RPMI 1640 supplemented with 10% FBS, L-glutamine, penicillin, streptomycin and 2-mercaptoethanol) with soluble anti-CD3 (145-2C11), anti-CD28 (37.51, 1 µg/mL), CTLA4-Ig (5 µg/mL), control human IgG (5 µg/mL), rapamycin (Calbiochem; 10 ng/mL in DMSO), or rmIL-2 (Roche). For anergy assays, primed CD4+ cells were rested for 24 hours in medium, purified, and restimulated for 18 hours with anti-CD3 and anti-CD28 immobilized on flat-bottom plates. DNA synthesis was measured by the addition of bromo-deoxyuridine (BrdU, Sigma) 6 hours before harvest at a final concentration of 10 µM.

### Flow Cytometry

Lymphocytes (0.5–1×10^6^) from primed cultures were stained with PerCP-conjugated anti-CD4 antibody (BD-Pharmingen) and PE-conjugated anti-Thy1.2 antibody (BD-Pharmingen), and cell division was analyzed by CFSE dilution. The absolute number of cell divisions accumulated within the CD4+ T cell subset was calculated as previously described [18]. BrdU incorporation by individual cells was detected using a Phoenix Red-conjugated anti-BrdU antibody (Phoenix Flow Systems) after fixation and permeabilization (Fix & Perm, Caltag Laboratories). DNA content was measured by the addition of 7-AAD (25 µg/ml) to the fixed and permeabilized cells for 30 min. The above fluorescence parameters were assessed using a FACSCalibur flow cytometer (Becton-Dickinson).

### T Cell Purification

CD4+ T cells were purified by negative selection using Bio-Mag beads (Qiagen) with antibodies against I-A/E, CD19, CD16/32, CD11b and CD8 (BD-Pharmingen), or were purified by positive selection using CD4+ MACS beads (Miltenyi Biotech). CD8+ T cells were also purified by either negative selection, or positive selection as above. Regulatory T cells (Treg) were purified by positive selection using Miltenyi CD4+CD25+ purification kits.

### Measurement of Cytokine Secretion

IL-2 and IFNγ secreted into the supernatant were measured by ELISA following manufacturer’s instructions (eBiosciences). Briefly, ELISA plates were coated with capture antibody overnight at 4°C. Plates were washed the next day in PBS with 0.05% Tween-20, blocked for 1 hour at room temperature, then incubated with dilutions of culture supernatant, in triplicate. A standard curve generated by diluting recombinant mouse IL-2 was also included on each plate in triplicate. Plates were incubated with culture supernatants for 2 hours at room temperature and washed. Biotin-conjugated detection antibody was added to the plates for 1 hour at room temperature then washed. Avidin-HRP detection enzyme was added to the plates for 30 minutes then plates received a final eight washes. Tetramethylbenzidine (TMB) substrate solution was added to the plates for 15 minutes to visualize cytokine amounts then the reaction was stopped with 1M phosphoric acid. Plates were read on a BioRad microplate reader at 450 nm.

### Cardiac Transplantation

Briefly, fully MHC mismatched hearts from BALB/c donors (H-2^d^) were transplanted into the peritoneal cavity of female wild-type (WT) C57BL/6 (H-2^b^) or p27^kip1^−/− mice on the C57BL/6 background (H-2^b^). The donor aorta was anastomosed to the recipient abdominal aorta, and the donor pulmonary artery was sutured to the recipient inferior vena cava. Transplant recipients received combined costimulatory blockade as indicated, consisting of anti-CD154 mAb (MR1, BioExpress, 200 µg/mouse, i.v.) administered on the day of transplant, and CTLA4-Ig (BioExpress, 200 µg i.p.) administered intraperitoneally on days 0, 2, and 4 post-transplant. In separate experiments mice were administered rapamycin daily at a dose of 0.1 mg/kg body weight i.p., or a single i.p. dose of cyclosporine (10 mg/kg body weight). Graft function was monitored by abdominal palpation and grafts were harvested at the time of rejection or as indicated. Hearts were deemed rejected when cardiac contractility was no longer detected, and rejection was confirmed visually at the time of graft harvest.

### Immunohistochemistry

At time of harvest, portions of cardiac grafts were fixed in formalin for paraffin sectioning or in OCT compound for immunohistology. Hematoxylin and eosin-stained paraffin sections were evaluated using the International Society of Heart and Lung Transplantation (ISHLT) standardized criteria for grading of cardiac allograft biopsies. Immunohistologic labeling of cryostat sections was performed using monoclonal and polyclonal Abs to CD4, CD8 and Foxp3 and respective Envision kits (Dako).

### *In vitro* Mixed Lymphocyte Reaction

T cells were CFSE labeled and seeded at 1×10^5^ per well in 96-well round bottom dishes. Irradiated syngeneic or allogeneic T-depleted splenocytes were added to the wells at ratios from 1∶5 to 1∶40. Cells were cultured for 3 days to 5 at 37°C in a 7% CO_2_ environment. Proliferation of allogeneic T lymphocytes as determined by CFSE dilution was assessed by flow cytometry, and clonal expansion was determined by counting absolute numbers of divided alloreactive cells by flow cytometry using reference beads.

### *In vivo* Mixed Lymphocyte Reaction

As described previously [19], CFSE-labeled donor splenocytes from p27^kip1^−/− (H-2^b^) mice or p27^kip1^+/+(H-2^b^) mice were retro-orbitally injected into B6xDBA F1 (H-2^d/b^) recipient mice (20×10^6^ per recipient). Recipients were either administered i.v. anti-CD154 mAb (200 µg/mouse) on day 0 in combination with CTLA4-Ig (i.p. 200 µg/mouse) on days 0 and 2, or were treated with equivalent doses of control immunoglobulin. Recipients were sacrificed on day 3 and spleens were harvested for subsequent flow cytometric analysis. Donor cells were differentiated from recipient cells by staining for differences in H-2^d^ expression and the frequencies of CD4+ alloreactive donor cells was determined by gating on CD4+ T cells that had diluted their CFSE.

### *In vitro* Suppression Assay

MACS-purified CD4+CD25− T cells were CFSE labeled and seeded at 5×10^4^ per well in 96-well round bottom dishes. Irradiated syngeneic T-depleted splenocytes were added to the wells at 1×10^5^ per well along with 0.5 mg/ml soluble anti-CD3 mAb. Cells were cultured for three days alone, or in the presence of purified CD4+CD25+ Treg at the indicated ratios. After three days suppression of responder cell proliferation was determined by flow cytometrically assessing the degree of inhibition of CFSE dilution.

### Statistics

For graft survival, Kaplan-Meier survival graphs were constructed and long-rank comparison of the groups was used to calculate P values. For ELISPOT assays P values were calculated with the Student’s t-test. Significance in the Parent into F1 studies was determined with a paired one-tailed t-test. Statistical analyses were performed with Prism software (GraphPad Software, San Diego, CA). Differences were considered significant at p<0.05.

### Ethics Statement

All animal studies were performed in accordance with the protocols approved by The Children’s Hospital of Philadelphia Institutional Animal Care and Use Committee.

## Results

### p18^ink4c^ Negatively Regulates Early Cell Cycle Progression in Activated CD4+ T Cells

**Figure 1:**
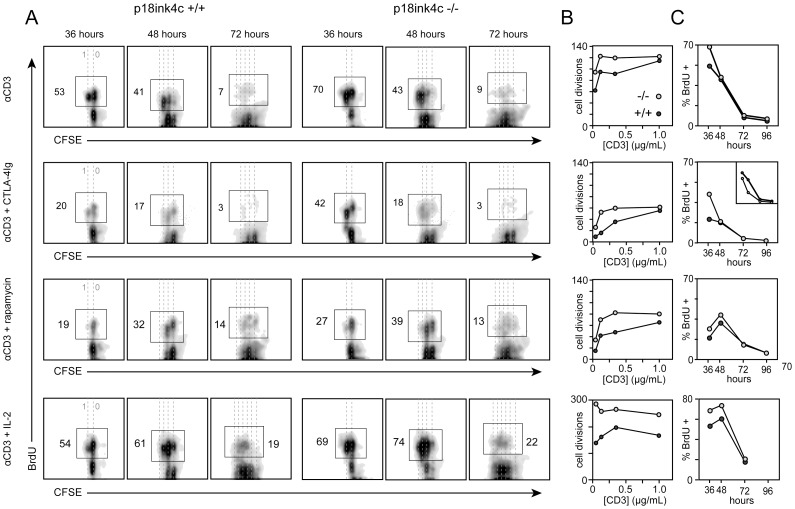
Kinetic analysis of DNA synthesis and cell division at the single-cell level in WT and p18^ink4c^-deficient CD4+ T cells. CFSE-labeled spleen and LN cells from p18^ink4c^+/+ (first three columns in **A**, dark gray circles in **B** and **C**) or p18^ink4c^−/− (last three columns in **A**, light gray circles in **B** and **C**) mice were stimulated *in vitro* with soluble anti-CD3 alone (top row), or in combination with CTLA-4Ig (5 µg/mL, second row), or rapamycin (10 ng/mL, third row), or IL-2 (20 U/mL, bottom row). Cultures were subjected to 6-hour pulses of BrdU at 30, 42, 66, or 90 hours, and harvested at 36, 48, 72 or 96 hours, respectively. Non-BrdU-pulsed cultures were used to set the BrdU-positive gates at each timepoint. Dotted lines in **A** indicate discrete cell divisions, with undivided cells on the far right. Cumulative cell division by the CD4+ T cell subset in **B** was quantified as described previously (34), and **C** depicts the frequency of BrdU-positive cells within the CD4+ subset at each time point. The results are representative of 2–3 independent experiments.

Cultures of p18^ink4c^−/− T cells exhibit enhanced DNA synthesis in response to primary stimulation [17]. To explore at which point in the cell cycle p18^ink4c^ functions to regulate T cell proliferation, we stimulated cultures of p18^ink4c^+/+ and p18^ink4c^−/− splenic lymphocytes in vitro with soluble agonistic anti-CD3 Ab and measured the kinetics of DNA synthesis and mitosis over a four-day period. In WT cultures, half of the activated CD4+ T cells had entered S phase and begun to synthesize DNA by 36 hours after stimulation, and about half of these cells had undergone a single round of mitosis (**Fig. 1** **A**, top row). By 48 hours, most of these cells had divided once, and 40% were synthesizing DNA and progressing through their second round of mitosis. During the subsequent 24 hours, cells progressed through on average two more division cycles, and by 72 hours the majority of cells fell out of cycle, with less than 10% of the cells still synthesizing DNA (**Fig. 1** **A**, top row). By 96 hours very few cells continued to proliferate. CD4+ T cells from p18^ink4c^−/− mice exhibited enhanced cell cycle progression at 36 hours (**Fig. 1** **A**, top row), with 35% more cells synthesizing DNA and many more of cells having undergone their second mitosis as compared with wild-type cells. This resulted in most of the p18^ink4c^-deficient cells having undergone an additional round of mitosis compared to WT cells by 48 hours (**Fig. 1** **A** and **C**, top row). However, throughout the rest of the response (from 48 to 96 hours), p18^ink4c^−/− and WT CD4+ T cells cycled at comparable rates (**Fig. 1** **A** and **C**, top row). The enhanced proportion of cells undergoing the first G1 to S phase transition in p18^ink4c^−/− cultures resulted in increased clonal expansion by the CD4+ T cell subset, especially under conditions of suboptimal TCR stimulation (**Fig. 1** **C**, top row). This suggests that p18^ink4c^ predominantly opposes G1 to S phase progression during the first cell division cycle in activated T cells.

CD28 costimulation is required for efficient IL-2 production and clonal expansion by T cells. Our previous studies showed that cultures of p18^ink4c^−/− T cells exhibit enhanced DNA synthesis in the absence of CD28 costimulation [17]. To further explore how p18^ink4c^ might act downstream of CD28, we stimulated WT and p18^ink4c^-deficient lymphocytes in the presence of CTLA4-Ig, which blocks B7-CD28 interactions. Consistent with our previous studies [13], the frequency of WT CD4+ T cells entering S phase and undergoing the first mitosis during the first 48 hours of stimulation was reduced by over 60% in CTLA-4Ig-treated cultures compared to cultures stimulated in the presence of physiologic costimulation (**Fig. 1** **A** and **B**, second row). Blockade of CD28 costimulation resulted in nearly complete cessation of DNA synthesis and mitosis at later time points (**Fig. 1** **A, B** and **C**, second row), and by the end of the response lead to a 5-fold decrease in cumulative cell division by WT CD4+ T cells (**Fig. 1** **C**, second row). p18^ink4c^-deficient CD4+ T cells exhibited only a 40% reduction in DNA synthesis at 36 hours in response to costimulatory blockade (**Fig. 1** **A** and **B**, second row), which represented only a 10% reduction compared to WT cells receiving physiologic costimulation. However, p18^ink4c^-deficient cells showed no proliferative advantage at later time points (**Fig. 1** **A** and **C**, second row). This CD28-independent early cell cycle entry translated to a 2- to 3-fold increase in cumulative cell division by the p18^ink4c^-deficient CD4+ T cells compared to WT cells, implying that p18^ink4c^ acts during the first cell cycle to sense CD28 costimulatory signals.

### p18^ink4c^ Opposes T Cell Growth Factor Receptor Signal Transduction

**Figure 2:**
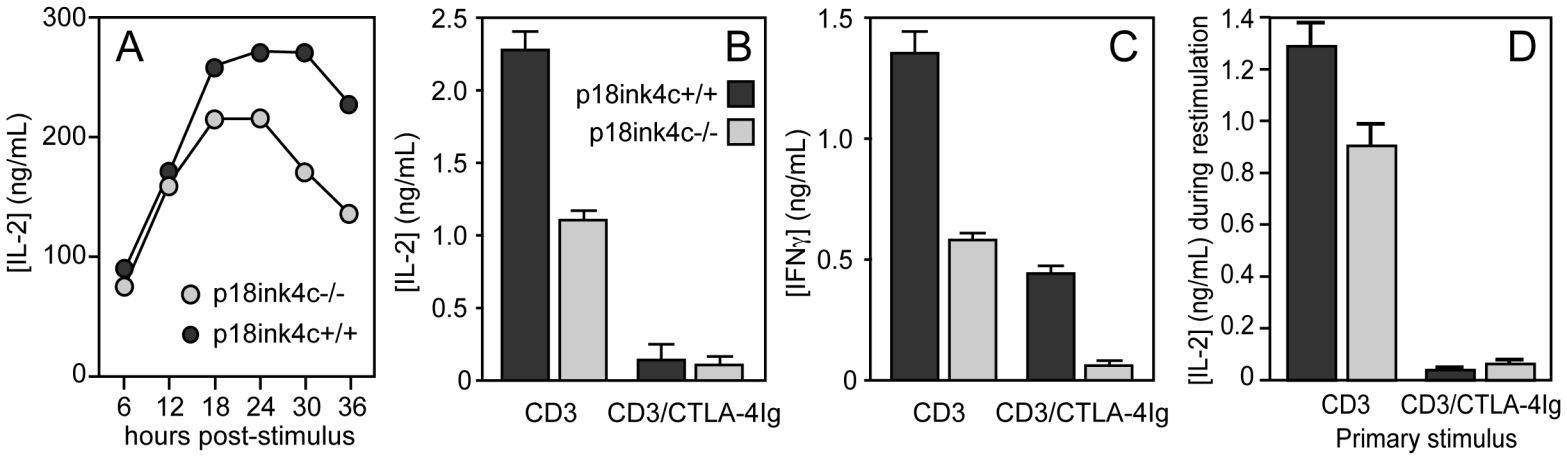
Reduced cytokine production by p18^ink4c^-deficient T cells. T cells from p18^ink4c^+/+ (dark gray symbols) or p18^ink4c^−/− (light gray symbols) mice were stimulated with soluble anti-CD3 Ab (5 µg/mL) or anti-CD3 plus CTLA-4Ig (10 µg/mL). IL-2 (**A** and **B**) and IFNγ (**C**) in the supernatants of primary cultures was measured by ELISA at the indicated time points in **A**, at 24 hours in **B**, and at 72 hours in **C**. CD4+ T cells purified from the primary cultures in **A–C** were rested, restimulated with plate-bound anti-CD3 (1 µg/mL), and IL-2 secretion was measured by ELISA at 18 hours post-stimulation (**D**). Data are plotted as the mean +/−SEM of duplicate cultures, and are representative of 2–3 independent experiments.

CD4+ T cells lacking p18^ink4c^−/− did not produce more IL-2 than WT cells (**Fig. 2** **A**), indicating that growth factor signaling may be augmented in the absence of p18^ink4c^. To test whether p18^ink4c^ acts downstream of growth factor signaling to regulate T cell proliferation, we stimulated WT and p18^ink4c^-deficient lymphocytes in the presence of rapamycin, an inhibitor of growth factor receptor-coupled PI3K/mTOR activation. As expected, treatment of WT CD4+ T cells with rapamycin resulted in a strong reduction of DNA synthesis and delay in S phase progression (**Fig. 1** **A** and **B**, third row). p18^ink4c^-deficient cells showed a similar delay in S phase progression (**Fig. 1** **A** and **B**, third row), but a moderate increase in the frequency of cells synthesizing DNA, and a 60–80% increase in cumulative cell divisions during the response (**Fig. 1** **C**, third row). These effects were not due to differential expression of IL-2 receptor, as both p18^ink4c^+/+ and p18^ink4c^−/− CD4+ T cells expressed equivalent levels of CD25, the a-chain of the high affinity IL-2 receptor (data not shown). Furthermore, we found that CD4+ T cells lacking p18^ink4c^ responded to the addition of exogenous IL-2 with augmented early DNA synthesis compared to WT cells (**Fig. 1** **A** and **B**, bottom row), as well as increased cumulative cell division at day 3 (**Fig. 1** **C**, bottom row). These data demonstrate that p18^ink4c^ modulates the sensitivity of T cells to mitogenic signals from CD28 and IL-2. Specifically, p18^ink4c^ appears to govern entry into the cell cycle, constraining T cell clonal expansion by limiting the proportion of activated cells entering the cycling pool.

### p18^ink4c^ Promotes Cytokine Production, but is Dispensable for Anergy in CD4+ T Cells

The results above demonstrate that p18^ink4c^ acts downstream of mitogenic signals to restrict T cell cycle progression, particularly under conditions of suboptimal mitogenic signals. T cell proliferation in the *in vitro* culture system utilized in these studies depends primarily on autocrine IL-2. We found that, instead of producing increased amounts of IL-2, p18^ink4c^−/− cultures accumulated less IL-2 in the supernatant than WT cultures during the first 36 hours of activation under physiologic costimulatory conditions (**Fig. 2** **A** and **B**). p18^ink4c^-deficient CD4 cells also produced less IFNγ than WT cells, particularly during primary stimulation under costimulatory blockade (**Fig. 2** **C**). These data indicate that p18^ink4c^ is somehow involved in TCR-coupled cytokine gene expression.

Anergy induction in several models has been linked to elevated expression of multiple cyclin-dependent kinase inhibitors [13], [20]. Previous studies have shown that the CDK inhibitory protein p27^kip1^ is required for anergy induction *in vitro* [13], [21], and for tolerance induction *in vivo* [15], [22]. Therefore, we hypothesized that p18^ink4c^ may also be required for anergy induction in CD4+ T lymphocytes. To test this, we primed WT or p18^ink4c^−/− T cells in the presence of CD28 costimulation to generate effector cells, or in the absence of CD28 costimulation to induce anergy. Both WT and p18^ink4c^-deficient CD4+ T cells primed in the absence of costimulation were unable to produce IL-2 (**Fig. 2** **D**) or IFNγ (data not shown) upon restimulation. Therefore, expression of p18^ink4c^ in CD4+ T cells, unlike p27^kip1^, is not necessary for anergy induction.

### p18^ink4c^ is not Required for *in vitro* Regulatory T Cell Function

**Figure 3:**
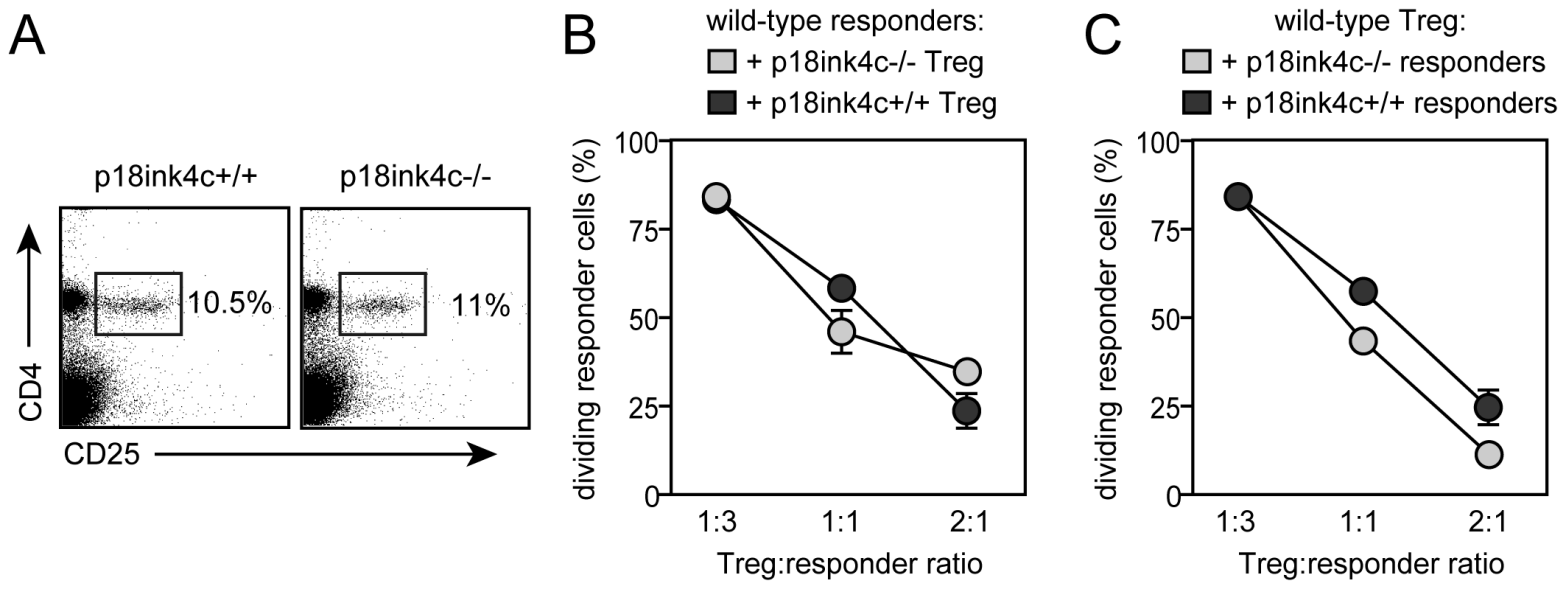
Frequency and function of regulatory T cells from p18^ink4c^−/− mice. Spleens from naïve, 4-week-old WT (left panel) and p18^ink4c^−/− (right panel) mice were stained for CD4 and CD25 to identify Treg (**A**). CFSE-labeled CD4+CD25− conventional T cells purified from wild-type mice were used as targets for CD4+CD25+ Treg purified from either WT (dark gray bars) or p18^ink4c^−/− (light gray bars) mice in a standard anti-CD3-stimulated suppression assay (**B**). CFSE-labeled CD4+CD25− conventional T cells purified from either WT (dark gray bars) or p18^ink4c^−/− (light gray bars) mice were used as targets for wild-type CD4+CD25+ Treg (**C**). Data are plotted as the mean +/−SD of triplicate cultures.

In addition to anergy and deletional mechanisms [23], tolerance to self and to organ transplants requires the activity of CD4+CD25+FoxP3+ Treg [24]. WT and p18^ink4c^-deficient mice exhibited similar frequencies of CD4+CD25+ cells in the spleen (**Fig. 3** **A**), indicating that p18^ink4c^ is not required for Treg development and homeostasis. To address whether p18^ink4c^ impacts Treg function, we purified CD4+CD25+ cells from WT and p18^ink4c^−/− mice and performed standard *in vitro* suppression assays. Both WT and p18^ink4c^-deficient Treg were able to suppress the proliferation of WT CD4+CD25− responder T cells with comparable efficiency (**Fig. 3** **B**), however conventional CD4+ T cells from p18^ink4c^-deficient were moderately, but consistently, more susceptible to Treg-mediated suppression than WT conventional cells (**Fig. 3** **C**). These results indicate that Treg do not require p18^ink4c^ for their development or function, but this CDK inhibitory protein actively contributes to Treg-mediated suppression of conventional T cell clonal expansion.

### p18^ink4c^ Promotes T Cell-mediated Alloimmune Responses

**Figure 4:**
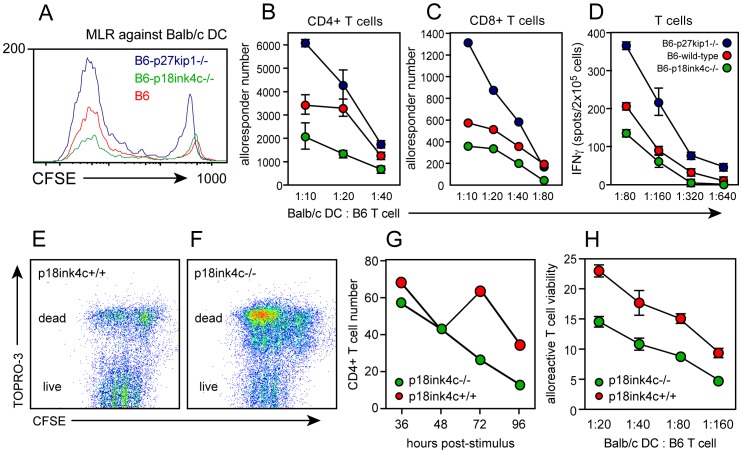
Effect of CDK inhibitor deficiency on *in vitro* alloimmune responses. CFSE-labeled lymphocytes from B6 WT (red symbols), p27^kip1^−/− (blue symbols), or p18^ink4c^−/− (green symbols) mice were cultured with irradiated DC derived from BALB/c bone marrow at the indicated T cell-to-DC ratios for 5 days. Alloresponsive cells were identified by dilution of CFSE (**A**), and the alloresponsive CD4+ T cells (**B**) and CD8+ T cells (**C**) were enumerated by flow cytometry using reference beads. The frequency of allospecific IFNγ-producing cells (**D**) was assessed at day 3 by replating responders and BALB/c DC in ELISPOT cultures overnight. CD4+ T cell viability was assessed by flow cytometry using the vital dye TOPRO-3 (34) in cultures stimulated *in vitro* with anti-CD3 Ab (**E–G**) or with allogeneic DC (**H**). Data are plotted as the mean +/−SEM of duplicate cultures, and are representative of 2–3 independent experiments.

Our data indicate that polyclonal T cell cycle progression and pro-inflammatory cytokine production are dysregulated in the absence of the D-type cyclin-dependent kinase inhibitory protein p18^ink4c^. To test whether alloimmune T cell responses are likewise regulated by p18^ink4c^, we stimulated B6 (H-2^b^) p18^ink4c^-deficient or WT T cells *in vitro* with BALB/c (H-2^d^) dendritic cells (DC), and quantified the alloreactive cell response on a single-cell level as a function of both cell division and IFNγ production. WT mixed lymphocyte reactions (MLR) showed strong expansion of alloreactive T cells (**Fig. 4** **A**, red histogram) of both the CD4 (**Fig. 4** **B**, red symbols) and CD8 (**Fig. 4** **C**, red symbols) subsets, which were able to differentiate and produce IFNγ when restimulated with BALB/c DC (**Fig. 4** **D**, red symbols). Consistent with previous studies (13, 15), MLR cultures from mice genetically deficient for p27^kip1^ showed enhanced alloreactive expansion and IFNγ production (**Fig. 4** **A–D**, blue symbols). However, while p18^ink4c^-deficient T cells underwent as many or more cell divisions as WT or p27^kip1^-deficient cells (**Fig. 4** **A**, green histogram), p18^ink4c^-deficient cultures failed to accumulate alloresponsive CD8+ and especially CD4+ cells compared to WT cultures (**Fig. 4** **B** and **C**, green symbols), and fewer of these cells were able to differentiate into IFNγ-producing effectors (**Fig. 4** **D**, green symbols). The use of vital dyes allowed us to track the fate of the responding T cells in our *in vitro* experiments by flow cytometry (**Fig. 4** **E** and **F**). This approach revealed that T cells dividing in the absence of p18^ink4c^ were more likely to die than WT T cells, in response to both mitogenic antibody (**Fig. 4** **G**, green symbols) and allogeneic DC (**Fig. 4** **H**, green symbols) stimulation. These data suggest that the reduced accumulation of effector T cells in p18^ink4c^-deficient cultures is due to an enhanced rate of apoptosis.

**Figure 5:**
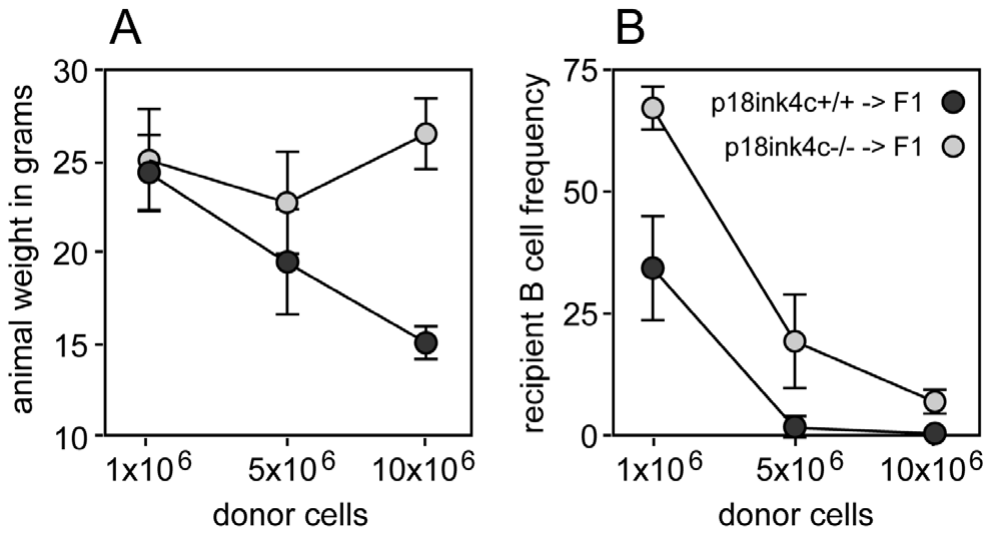
Effect of p18^ink4c^ deficiency on the development of graft-vs.-host disease. C57BL/6 X BALB/c F1 mice aged 4–6 weeks were subjected to irradiation (600 cGy) 6 hours before adoptive transfer of 10×10^6^, 5×10^6^ or 1×10^6^ B6 WT (dark gray symbols, n = 5) or p18^ink4c^−/− (light gray symbols, n = 5) donor splenocytes. Recipient mice were weighed daily and observed for outward signs of GVHD. On day 13, recipient mice were weighed (**A**) and sacrificed to determine the frequency of host CD19+ B lymphocytes in the spleen by flow cytometry (**B**).

To test whether p18^ink4c^ influences T cell-mediated alloimmunity *in vivo*, we utilized an acute graft-vs.-host disease (GVHD) model. GVHD is an inflammatory disease mediated by pro-inflammatory cytokines produced by alloreactive CD4+ T cells, as well as solid organ damage and ablation of host hematopoietic cells by alloreactive CD8+ CTL. For these experiments, age- and sex-matched C57BL/6 X BALB/c F1 (H-2^d/b^) mice were conditioned with sublethal irradiation (600 cGy), and graded doses of p18^ink4c^−/− or p18^ink4c^+/+ C57BL/6 (H-2^b^) splenocytes were adoptively transferred. As early as one week post-transfer, recipients of wild-type donor cells began to appear hunched and cachectic, and recipients of higher donor cell numbers experienced significant weight loss by 2 weeks post-transfer (**Fig. 5** **A**, dark grey symbols). Recipients of 5 or 10 million WT donor cells also showed signs of massive hematopoietic cell ablation, as seen by the lack of peripheral host B cells in the spleen (**Fig. 5** **B**, dark grey symbols), while recipients of 10^6^ WT donor cells contained only half the normal frequency of peripheral B cells (**Fig. 5** **B**, dark grey symbols). Conversely, recipients of even the highest dose of p18^ink4c^-deficient donor cells maintained a healthy appearance throughout the experiment and did not lose weight by 2 weeks (**Fig. 5** **A**, light grey symbols). These mice also showed signs of much milder host hematopoietic cell ablation. Recipients of 10^6^ p18^ink4c^−/− donor cells had normal frequencies of peripheral B cells (**Fig. 5** **B**, light grey symbols), and while recipients of higher p18^ink4c^−/− donor cell doses did experience significant host B cell loss, the B cell frequencies in these animals were 10- to 20-fold higher than the B cell frequencies in recipients of equal numbers of WT donor cells (**Fig. 5** **B**, light grey symbols). These data demonstrate that alloreactive T cells must express p18^ink4c^ in order to mediate significant GVH disease *in vivo*.

### p18^ink4c^ Opposes Acquired Transplantation Tolerance

We previously established that the CDK inhibitor p27^kip1^ acts as a checkpoint telling CD4+ T cells whether they have received the appropriate costimulatory and growth factor signals to progress into the cell cycle and expand. Without p27^kip1^, T cells exhibit enhanced alloresponses, anergy cannot be induced in CD4+ T cells by costimulation blockade *in vitro*, and costimulatory blockade fails to induce transplantation tolerance in mice lacking p27^kip1^ [13], [15]. Our current data demonstrate that, conversely, p18^ink4c^ is not required for anergy induction, and instead, expression of p18^ink4c^ appears to be necessary for efficient T cell survival, cytokine production and alloimmune responses.

**Figure 6:**
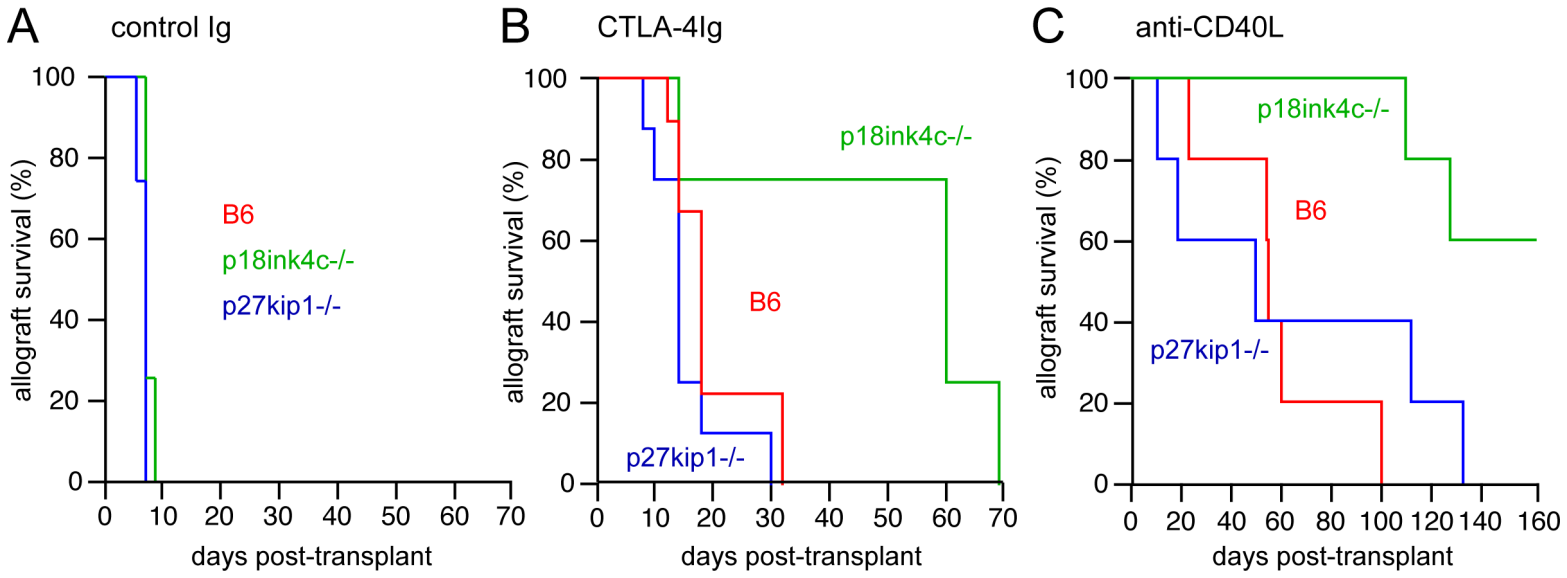
Effect of CDK inhibitor deficiency on cardiac allograft rejection. Kaplan-Meier survival curves for B6 (red lines), p27^kip1^−/− (blue lines) and p18^ink4c^−/− (green lines) B6 recipients of BALB/c heterotopic cardiac allograft treated with control Ig (**A**, 200 µg i.p. on days 0, 2, and 4), CTLA-4Ig (**B**, 200 µg i.p. on days 0, 2, and 4) or anti-CD154 mAb (**C**, MR1, 200 µg i.v. on day 0). Mean survival times (MST) for the control Ig groups are: B6 MST = 7, n = 3; p27^kip1^−/− MST = 7, n = 3, p18^ink4c^−/− MST = 7, n = 3. MST for the CTLA-4Ig groups are: B6 MST = 18 days, n = 9; p27^kip1^−/− MST = 17 days, n = 5; p18^ink4c^−/− MST = 60 days, n = 4, p<0.05. MST for the anti-CD154 groups are: B6 MST = 55 days, n = 5, p27^kip1^−/− MST = 57 days, n = 5, p18^ink4c^−/− MST>135 days, n = 5, p<0.05.

To test whether p18^ink4c^ plays a role, either negative or positive, in T cell-mediated allograft rejection, we utilized a murine model of fully MHC-mismatched cardiac transplantation. With no treatment, both p18^ink4c^-deficient and p27^kip1^-deficient B6 recipients rejected WT BALB/c cardiac allografts in 6–8 days with comparable kinetics to WT mice (**Fig. 6** **A**), indicating that p18^ink4c^ is not required for acute rejection of fully mismatched cardiac allografts. Blockade of B7-CD28 interactions *in vivo* with CTLA4-Ig prolonged allograft survival in WT B6 recipients by approximately 10 days (**Fig. 6** **B**, red line, MST = 18 days, n = 9). Consistent with our previous study [15], genetic elimination of p27^kip1^ resulted in slightly accelerated graft rejection (**Fig. 6** **B**, blue line, MST = 14 days, n = 8). Conversely, the majority of p18^ink4c^-deficient mice treated with CTLA4-Ig accepted cardiac allografts for a full 2 months, with rejection occurring shortly thereafter (**Fig. 6** **B**, green line, MST = 60 days, n = 4). Blockade of the CD40 costimulatory pathway with anti-CD154 (MR1) caused marked prolongation of allograft survival in both wild-type (**Fig. 6** **C**, red line, MST = 55 days, n = 5) and p27^kip1^-deficient (**Fig. 6** **C**, blue line, MST = 57 days, n = 5) recipients, but induced long-term allograft acceptance in p18^ink4c^−/− mice (**Fig. 6** **C**, green line, MST>135 days, n = 5). In addition, p18^ink4c^−/− mice experienced prolonged allograft survival in response to treatment with rapamycin (0.1 mg/kg daily, MST = 32 days) or cyclosporine (single dose of 10 mg/kg, MST = 22 days) compared to WT recipients (MST = 10 days for both treatments) (data not shown). The results from both the cardiac allograft and GVHD models together suggest that p18^ink4c^ promotes T cell effector function, particularly under conditions of suboptimal antigenic or costimulatory signals.

## Discussion

p18^ink4c^ is a protein of 18 kD first identified through its ability to bind CDK6 in a yeast two-hybrid screen [25]. Like other members of the ink4 family, p18^ink4c^ has a tertiary structure consisting of repeating helix-turn-helix units and ankyrin repeats, a motif commonly utilized in protein-protein interactions [26]. p18^ink4c^ binds strongly to CDK6, with weaker binding to the D-type cyclin-dependent kinase CDK4, and no binding to CDK2 [3], leaving p18^ink4c^-CDK6-cyclin D3 as the major G1 regulatory complex in lymphocytes [17], [27]. Mice genetically deficient for p18^ink4c^ were originally reported to exhibit gigantism, organomegaly, and hyperplasia of the spleen and thymus [28], with p18^ink4c^-deficient CD3+ T cells exhibiting a 4-fold increase in thymidine incorporation when stimulated *in vitro* with anti-CD3 antibodies [17]. Our studies extend these findings, establishing that p18^ink4c^ helps to regulate early activation and cell cycle progression, but does not contribute significantly to later cell divisions. Unlike p27^kip1^, which is targeted for proteolytic degradation by mitogenic signals, p18^ink4c^ protein levels remain constant during the first 36 hours after stimulation of quiescent T lymphocytes [17], and do not change substantially over a three day period of activation (our unpublished observations). The first cell division following activation of quiescent (G_0_) T lymphocytes requires approximately 36 hours [18], corresponding to the time it takes for D cyclins to be synthesized, assembled with their CDK partners, and transition from G1 through S phase. During subsequent divisions, T cells do not re-enter G_0_ and spend very little time in G_1_ phase [13], [29]. Deletion of cyclin D1 or CDK4 in mouse embryonic fibroblasts (MEF) causes a delay in G_0_ to G_1_ progression, but has minimal effect on continuously cycling MEF [30], [31]. Also our results indicate that costimulatory blockade and mTOR inhibition largely do not require the activity of p18^ink4c^ for their cell cycle inhibitory effects. Together, these data suggest that T cells are not heavily dependent upon the D-type cyclin-CDK6-ink4 pathway for prolonged clonal expansion.

While p18^ink4c^ is clearly a negative regulator of early T cell cycle progression, our study shows that this protein also acts as a positive regulator of T cell differentiation. While this seems initially paradoxical, p18^ink4c^ has been shown to regulate cellular differentiation in several tissues where cell fate is linked to cell division. For instance, B lymphocyte activation is normally accompanied by a phase of clonal expansion, followed terminal differentiation into non-proliferative, antibody-secreting plasma cells. However, B cells deficient for p18^ink4c^ are hyperproliferative and fail to undergo terminal differentiation, leading to a severe defect in antibody responses [32]. p18^ink4c^ also promotes the differentiation of hematopoietic stem cells by limiting self-renewal divisions in the primitive cell pool [33]. We have found that CD69 upregulation, MAPK activation, and IκBα degradation occur to a similar degree in wild-type and p18^ink4c^-deficient cells (our unpublished observations). Further studies will be required to determine if other TCR- or cytokine-coupled pathways involved in T cell differentiation are affected by p18^ink4c^. During muscle development, immature myoblasts undergo a p18^ink4c^-dependent cell cycle arrest as they differentiate into myotubes. In the absence of p18^ink4c^, differentiating myoblasts continue to proliferate and die by apoptosis [34]. CDK activity is known to induce the transcription factor E2F1 [35], [36], which promotes apoptosis through stabilization of p53 and p73 [37]–[41]. We likewise observed an increased rate of apoptosis in activated p18^ink4c^-deficient T cells, suggesting that dysregulated CDK activity in these cells may lead to apoptosis of differentiated effector cells. We find that p18-deficient and wild-type T cells are equally susceptible to active death domain signaling through Fas, TNF and redox imbalance (our unpublished observations), suggesting that p18^ink4c^ may operate to block intrinsic cell death mechanisms involving p53 family members, but more studies will be required to understand how p18^ink4c^ controls T cell survival and function.

Our results demonstrate that the D-type CDK inhibitor p18^ink4c^ contributes to alloimmune T cell differentiation and function, and is required for graft-vs.-host disease and costimulation-resistant allograft rejection. Interestingly, this phenotype is opposite from mice lacking the E-type CDK inhibitor p27^kip1^, which are resistant to the induction of tolerance [15]. Instead, p18^ink4c^-deficient mice resemble mice lacking CDK2, the target of p27^kip1^, which are more susceptible to costimulation blockade-induced tolerance [42]. These studies show that cyclin-dependent kinases and their inhibitors play important and complex roles in regulating T cell effector function, and may therefore represent novel immunomodulatory targets. However, in order to use cell cycle regulatory proteins as therapeutic targets for immunopathologic disease, a more complete understanding of their function will be required.
